# Polyphenol-based modulation of the Glo1–Nrf2–RAGE axis in diabetes and neurodegeneration: mechanistic evidence, translational constraints, and critical appraisal

**DOI:** 10.3389/fphar.2026.1877182

**Published:** 2026-07-20

**Authors:** Adnan Amin, Graciela Dolores Ávila-Quezada

**Affiliations:** 1 Department of Life Sciences, Yeungnam University, Gyeongsan, Republic of Korea; 2 Facultad de Ciencias Agrotecnológicas, Universidad Autónoma de Chihuahua, ChihuahuaChih, Mexico

**Keywords:** flavonoids, methylglyoxal, neurodegeneration, Nrf2, oxidative stress

## Abstract

The key pathological mechanisms in diabetes and neurodegeneration generally involve a progressive accumulation of reactive carbonyl species (RCS), accompanied by increased oxidative stress and the accumulation of inflammatory mediators. Thus, a clear understanding of the Glo1–Nrf2–RAGE axis is crucial as it plays a key role in redox imbalance, regulation of cell responses to methylglyoxal (MG)-induced glycation, and chronic inflammation. Even though glyoxalase I (Glo1) detoxifies MG to mitigate advanced glycation end product (AGE) formation, the transcription of antioxidant enzymes by nuclear factor erythroid 2–related factor 2 (Nrf2) is reactivated. Conversely, persistent activation of the receptor for AGE (RAGE) further amplifies inflammatory cascades and tissue damage. A continuous dysregulation of “this axis” can contribute to the pathogenesis of several complications, including diabetes and neurodegeneration. Nevertheless, polyphenols have emerged as nutraceutical candidates that may modulate the Glo1–Nrf2–RAGE axis due to their specialized structural features. Key polyphenols, such as quercetin, resveratrol, curcumin, epigallocatechin gallate, luteolin, and apigenin, enhance Glo1 expression and activity, promote Nrf2 nuclear translocation via Keap1 modification, and lower RAGE expression and ligand binding. However, numerous challenges, such as limited bioavailability, metabolic instability, and “interindividual variability,” hinder their clinical translation. We have tried to fill the research gap by combining recent evidence from preclinical, clinical, and molecular studies, with the aim of highlighting the pleiotropic effects of these metabolites. In addition, the molecular effects of polyphenolics with reference to modulation of mitochondrial function, regulation of epigenetic mechanisms, and interactions with the gut–brain axis are detailed.

## 1 Introduction

At the cellular level, the Glo1–Nrf2–RAGE axis integrates Keap1-mediated redox, electrophile sensing, RAGE-mediated ligand recognition, Nrf2-dependent antioxidant transcription, Glo1-mediated MGO detoxification and RAGE-associated inflammatory signaling (Michalak and Michalak, 2025; Ali et al., 2026). The Glo1 is considered to catalyze the rate-limiting step of the glyoxalase system, detoxifying RCS, including MG, thereby preventing interaction with nucleophilic macromolecules (Alhujaily, 2024). The Nrf2 is primarily involved during such an enzyme-based protection (Rabbani and Thornalley, 2022; Xue et al., 2025). Nrf2 is retained within the cytoplasm by Kelch-like ECH-associated protein 1 (Keap1) during normal (healthy) conditions (Mukherjee and Gopalakrishnan, 2023). However, substantial dissociation and nuclear translocation are observed during oxidative or electrophilic stress, which activates redox homeostasis-related genes, including *Glo1* (Ngo and Duennwald, 2022). Accumulation of AGEs and damage-associated molecular patterns can activate RAGE and amplify oxidative and inflammatory signaling (Sun et al., 2025). Together, these processes influence cellular capacity to maintain redox balance, limit carbonyl stress, and respond to metabolic and inflammatory injury (Triposkiadis et al., 2022; Dharshini et al., 2023).

Methylglyoxal is considered a focal RCS since it generally links altered glucose metabolism with glycation, oxidative stress, and inflammatory signaling (Nigro et al., 2017). As a highly reactive dicarbonyl generated mainly from glycolytic intermediates, methylglyoxal is a major precursor of advanced glycation end products and contributes to dicarbonyl stress in diabetes, vascular injury, aging, and neurodegenerative disorders (Rabbani, 2022). Its detoxification depends primarily on the glyoxalase system, with Glo1 serving as the rate-limiting enzyme; therefore, methylglyoxal accumulation reflects both increased carbonyl formation and impaired detoxification capacity (Rabbani et al., 2016). In the nervous system, methylglyoxal-derived glycation is associated with mitochondrial dysfunction, proteostasis disruption, and AGE–RAGE-mediated neuroinflammation (Allaman et al., 2015). Thus, methylglyoxal provides a mechanistically justified entry point for evaluating polyphenol-mediated modulation of the Glo1–Nrf2–RAGE axis.

With aging, several chronic disorders, particularly those associated with oxidative damage and impaired detoxification capacity, are linked to dysregulation of the axis (Syed et al., 2023). With advancing age, mitochondrial function and antioxidant defenses decline markedly, accompanied by elevated carbonyl and RAGE expression, low Glo1 activity, and diminished Nrf2 responsiveness (Aragonès et al., 2021; Samanta et al., 2025). Consequently, impaired regenerative capacity, tissue degeneration, and cell senescence can occur (Samanta et al., 2025). In diabetes, persistent hyperglycemia accelerates Glo1 dysfunction and elevates MG formation (Syed et al., 2023). This progression increases AGE accumulation, vascular dysfunction, and multiorgan injury through RAGE-associated inflammatory signaling, oxidative stress, and related metabolic disturbances (Vianello et al., 2025). Comparable mechanisms, including carbonyl stress, mitochondrial dysfunction, impaired proteostasis, and neuroinflammation, are also implicated in neurodegenerative disorders such as amyotrophic lateral sclerosis, Alzheimer’s disease, and Parkinson’s disease (Henry et al., 2025).

Polyphenols, abundant in fruits, vegetables, herbs, and teas, are structurally diverse phytochemicals (Maniglia et al., 2021; Rana et al., 2022). Substantial research has examined polyphenols as potential modulators of the Glo1–Nrf2–RAGE axis because of their effects on redox signaling, carbonyl stress, and inflammation (Wani et al., 2023). Their direct interaction with Keap1 cysteine residues releases Nrf2, mainly due to their redox-active nature (Kuczyńska et al., 2022). Nrf2, in turn, transcriptionally upregulates antioxidant and phase II detoxifying enzymes (Glo1) (Yadav et al., 2023). Various common polyphenols (from fruits and vegetables), including quercetin and epigallocatechin gallate (EGCG), augment Glo1 activity and the expression of its gene directly (Méndez-Líter et al., 2022; Zhou et al., 2025). Such an effect is accompanied by a suppression of RAGE expression and downstream “inflammatory signaling” in numerous cell systems (Mokra et al., 2022; Yang et al., 2024). Several mechanistic studies have shown that the bioefficacy of polyphenols is governed by metabolite-specific structure–activity relationships, their extensive tissue distribution, and the disease context, underscoring the need for precise pharmacological characterization. Despite the pronounced effects of polyphenols in the Glo1–Nrf2–RAGE axis, a few constraints regarding their bioavailability, including limited aqueous solubility, instability, and first-pass metabolism, exist (Datta et al., 2026). These limitations have prompted interest in formulation strategies, including polymeric nanoparticles, liposomes, and self-emulsifying systems, to improve polyphenol stability, bioavailability, and tissue delivery. Based on the polyphenol profiles, polymeric nanoparticles, liposomes, and self-emulsifying systems are of particular interest and have potential implications (Truzzi et al., 2023).

Although several independent reviews have discussed polyphenols in relation to Nrf2, a receptor for RAGE, and metabolic or neurodegenerative disorders, these pathways are often evaluated separately (Ma, 2013). In contrast, evidence indicates that Glo1, Nrf2, and RAGE form an interconnected regulatory axis linking MG detoxification, oxidative stress responses, and inflammatory signaling (Rabbani and Thornalley, 2011). Therefore, this review integrates these metabolites into a mechanistic framework and evaluates how polyphenols concurrently regulate carbonyl stress, redox adaptation, and AGE–RAGE-mediated inflammation. Its novelty lies in its axis-based interpretation of polyphenol activity. Previous reviews have examined polyphenols in relation to Nrf2 signaling, RAGE activation, diabetes, or neurodegeneration, often as separate topics (González et al., 2020; Bhattacharya et al., 2023). However, these have not systematically integrated Glo1-mediated MG detoxification with Nrf2-dependent antioxidant regulation and RAGE-driven inflammatory signaling as a unified disease-relevant axis. The present review addresses this gap by framing the Glo1–Nrf2–RAGE axis as an interconnected regulatory network linking carbonyl stress, oxidative imbalance, and chronic inflammation. Such an axis-based perspective provides a mechanistic basis for evaluating polyphenols as multitarget modulators rather than general antioxidants or isolated pathway-specific agents. By applying this framework to diabetes and neurodegeneration, the review adds a conceptual and translational synthesis that extends beyond prior pathway-focused literature.

## 2 Literature search strategy

Botanical names cited in this review were checked against Kew’s Medicinal Plant Names Services and Plants of the World Online. Accepted species names, authorities, families, and pharmacopoeial drug names, where assigned, were recorded for plant-derived extracts and preparations discussed in the reviewed studies. These details are provided in the Supplementary Table. This review was developed employing a structured narrative survey of the literature on the Glo1–Nrf2–RAGE axis and its modulation by polyphenols. Relevant studies from PubMed, Scopus, and Web of Science (2000–2026) were collected, with emphasis on recent mechanistic and translational evidence. Search combinations included “glyoxalase one methylglyoxal detoxification,” “Nrf2–Keap1 antioxidant response,” “AGE–RAGE signaling inflammation,” “polyphenols Glo1 regulation,” “polyphenols Nrf2 activation,” and “polyphenols RAGE inhibition in diabetes or neurodegeneration.” Primary studies providing mechanistic or experimental evidence cell, animal, and clinical were included, while reviews were selectively applied for contextual integration. Studies were excluded when they lacked relevance to the review axis, focused on unrelated phytochemical effects, or did not provide sufficient experimental or clinical detail. The final literature set was curated based on its relevance to axis-level interactions among Glo1, Nrf2, and RAGE and their modulation by polyphenols. Based on their nature (narrative approach), reviews with heterogeneous or context-dependent outcomes, particularly in clinical settings, were considered relevant. To improve interpretability of the experimental evidence, studies were categorized as *in vitro*, *in vivo*, or clinical. For studies involving plant extracts, polyphenol-rich preparations, or nutraceutical formulations, reporting quality was considered according to ConPhyMP best-practice principles. As this is a narrative review and does not report original extract preparation or chemical analysis, items related to voucher deposition, extraction procedures, batch production, and primary phytochemical profiling were treated as not applicable.

## 3 The glo1–nrf2–rage axis: A molecular overview

Having outlined the molecular architecture of the Glo1–Nrf2–RAGE axis, this section evaluates how polyphenols interact with each of its regulatory nodes. This axis characterizes a firmly regulated molecular interface between oxidative stress, glycation, and inflammatory signaling (Yue et al., 2022), and an overview is presented below.

### 3.1 Glyoxalase system and the role of Glo1

The cell glyoxalase system is a crucial evolutionarily conserved enzymatic defense mechanism that primarily detoxifies α-oxoaldehydes (Scirè et al., 2022; Vašková et al., 2025). In this two-step system of enzyme–enzyme interaction, Glo1 is the first and rate-limiting enzyme. It primarily catalyzes the spontaneous isomerization of the hemithioacetal produced by a reaction between MG and GSH into S-D-lactoylglutathione (Kalapos et al., 2022; Campos Campos, 2024), which is then converted to D-lactate by Glo2 (Yang et al., 2025). The maintenance of carbonyl and redox homeostasis generally relies on the expression and catalytic efficiency of Glo1, which is sensitive to cell redox conditions and GSH availability (Garcia Cortizo, 2024). Under physiological conditions, Glo1 limits the accumulation of reactive dicarbonyl metabolites, including methylglyoxal, glyoxal, and 3-deoxyglucosone (Rabbani et al., 2016). Methylglyoxal is emphasized here because it is a glycolysis-derived Glo1 substrate and a major precursor of advanced glycation end products, linking carbonyl stress to AGE–RAGE-mediated oxidative and inflammatory signaling (Allaman et al., 2015; Borysiuk et al., 2022). This readily modifies various amino acid residues within proteins, including arginine, lysine, and cysteine, and functionally impairs the formation of AGEs (Pucci et al., 2021). Multiple complicated cell mechanisms, including GSH depletion, reduced transcriptional activity, and post-translational modifications, including nitrosylation and acetylation, downregulate *Glo1* (Amaro et al., 2026). In several established models, experimentally reduced Glo1 activity generally correlates with elevated MG levels and AGE accumulation (Berdowska et al., 2023; Alhujaily, 2024). Moreover, *Glo1* expression is modulated by key transcriptional regulators, including HIF-1α, Nrf2, and AP-1, in response to metabolic and oxidative stress (Belanova et al., 2022). Such dynamic regulation of Glo1 signifies a metabolic barrier, and its dysfunction increases the AGE burden.

### 3.2 Nuclear factor erythroid 2–Related factor 2 (Nrf2)

Among the members of the Cap’n’Collar (CNC) leucine zipper family, Nrf2 is a vital redox-sensitive transcription factor (Zgorzynska et al., 2021) with a pivotal role in cytoprotection against oxidative, electrophilic, and xenobiotic stresses (Sekine and Motohashi, 2021). Under basal conditions, Nrf2 is sequestered within the cytoplasm via its interaction with Keap1, thereby promoting proteasomal degradation (Shilovsky and Dibrova, 2023). However, in response to oxidative or electrophilic stress, critical cysteine residues on Keap1 are modified, stabilizing Nrf2 and promoting its nuclear translocation (Song et al., 2024), where it binds to antioxidant response elements (AREs) within the promoters of target genes. A molecular cascade initiates, the transcription of a battery of cytoprotective enzymes, including heme oxygenase-1 (HO-1), NAD(P)H:quinone oxidoreductase 1 (NQO1), glutamate–cysteine ligase, and notably, Glo I (Alonso-Piñeiro et al., 2021; Hayes et al., 2025). In the context of metabolic dysregulation—increased carbonyl stress—the role of Nrf2 gains particular significance in regulating Glo1 (Luchkova et al., 2024). Glo1 activates Nrf2, thereby enhancing the steady-state clearance of MG from cells (Syed et al., 2023; Liu et al., 2024), and promotes mitochondrial biogenesis, which in turn reduces RAGE-mediated proinflammatory signaling (Guarneri et al., 2021; Liu et al., 2025). These findings position Nrf2 as a central regulatory node linking redox defense to anti-glycation and anti-inflammatory pathways.

### 3.3 Receptor for advanced glycation end products (RAGE)

RAGE is a pattern recognition receptor of the immunoglobulin superfamily (Cross et al., 2024). Among other roles, RAGE is primarily involved in the transduction of proinflammatory and pro-oxidant signals that enhance the accumulation of AGEs in cells (Dong et al., 2022). Regarding structure, RAGE has one transmembrane domain, an extracellular ligand-binding domain, and a cytoplasmic tail involved in the intracellular signaling system (Dong et al., 2022). Ligand–RAGE binding induces a series of conformational changes and further activates multiple downstream signaling cascades, including NF-κB, MAPKs, JAK/STAT, and PI3K/Akt (Dong et al., 2022), which upregulate several proinflammatory cytokines, adhesion molecules, and reactive oxygen species (ROS)-generating enzymes (Radziszewski et al., 2024). Thus, a self-sustaining inflammatory microenvironment is created that exacerbates cell dysfunction and tissue injury in chronic diseases. In addition to AGEs, various ligands like S100 proteins, HMGB1, and amyloid β-peptides bind to RAGE with robust affinity (Cross et al., 2024). Such multi-ligand interactions indicate a broader role for RAGE in sterile inflammation and other crucial pathways (Dong et al., 2022). In diabetes, for instance, elevated MG and AGE levels enhance RAGE expression, particularly in endothelial and renal tissues (Taguchi and Fukami, 2023), thus contributing greatly toward excessive vascular damage and nephropathy (Parwani and Mandal, 2023). Nevertheless, in neurodegenerative disorders, AGE–RAGE interactions within the brain promote neuroinflammation and amyloidogenesis (Bhattacharya et al., 2023). Notably, Glo1 activity serves as an upstream brake on RAGE-mediated inflammation by inversely regulating RAGE signaling (Wang et al., 2021; Bhattacharya et al., 2023).

### 3.4 Interconnected regulatory dynamics

**FIGURE 1:**
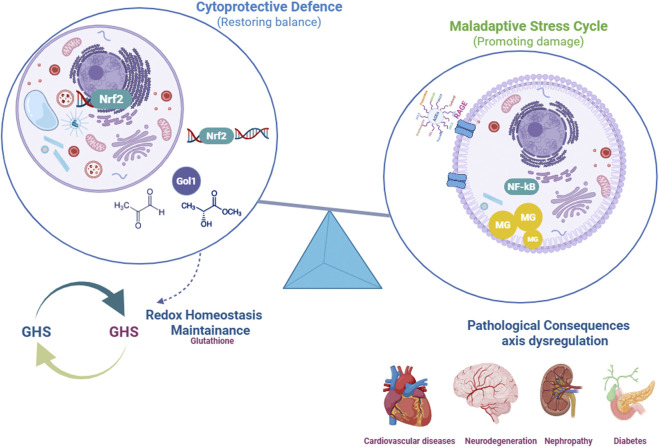
Functional balance of the Glo1–Nrf2–RAGE axis in cytoprotection and stress-associated disease progression. This figure illustrates how the Glo1–Nrf2–RAGE axis shifts between cytoprotective adaptation and maladaptive stress signaling. Nrf2-driven antioxidant responses and Glo1-mediated methylglyoxal detoxification support redox homeostasis, whereas AGE–RAGE activation amplifies inflammatory and oxidative injury under pathological conditions.

Maintenance of redox and metabolic equilibrium under physiological conditions is maintained through a dynamic interplay among Glo1, Nrf2, and RAGE, which reflects a finely tuned regulatory system (Wang et al., 2021; Li et al., 2025). However, during “pathological” or “diseased” conditions, including hyperglycemia, ischemia, or toxin exposure, such a system becomes “maladaptive” (Li et al., 2025). In such a state, reduced Nrf2 activity, impaired Glo1 function, and persistent RAGE activation occur, propagating oxidative and inflammatory damage (Manfredelli et al., 2025). More crucially, recent findings designate several pharmacological (e.g., polyphenolic) or nutraceutical agents (berries) that simultaneously enhance Nrf2 and Glo1 while suppressing RAGE (Cheng et al., 2025). An integration of these three nodes, i.e., Glo1, Nrf2, and RAGE, not only provides mechanistic insights into pathogenesis but also identifies strategic intervention points for therapeutic modulation (Figure 1).

## 4 Polyphenols as modulators of the glo1–nrf2–rage axis

Polyphenols have garnered significant attention for their ability to modulate intracellular redox signaling, metabolic detoxification pathways, and inflammation-related gene networks (Rudrapal et al., 2024). The varied bioactivities and differential target selectivity of polyphenols are mainly reliant on structural features such as −OH group positioning, conjugation, and ring-like structure (Halake et al., 2016). Evidence from *in vitro* cell systems and *in vivo* animal models indicates that polyphenols act not only through direct antioxidant activity or target binding but also through modulation of transcriptional and post-translational regulatory networks. Clinical evidence remains more limited and is therefore discussed separately in the disease-specific sections (Zagoskina et al., 2023).

Unless otherwise stated, compound-specific effects described in the following sections represent findings reported in the cited experimental systems and should not be interpreted as proof of selective target engagement or clinical efficacy. For compounds with known or plausible assay-interference liabilities, the strength of mechanistic inference depends on orthogonal validation, interference controls, physiologically relevant exposure, and confirmation *in vivo* or in humans, as summarized in Table 1 and discussed in Section 7.5.

**TABLE 1:** Assay-interference concerns and validation requirements for major polyphenols discussed in this review.

Compound	Key liability	Evidence required	Ref
Curcumin/curcuminoids	Instability, reactivity, aggregation, optical interference	Stability controls; orthogonal assays; target engagement; exposure matching	Nelson et al. (2017)
Quercetin	Fluorescence quenching, redox activity, metal chelation	Optical controls; non-optical confirmation; target engagement; metabolite testing	Dirimanov and Högger (2020)
EGCG	Auto-oxidation, quinone formation, protein reactivity	Oxidation controls; redox counter-screens; orthogonal assays; target engagement	Palhano et al. (2013)
Resveratrol	Membrane perturbation and low parent-compound exposure	Membrane-independent assays; target engagement; PK–PD confirmation	Magalhães et al. (2022)
Luteolin	Catechol-associated redox and chelation liability	Redox controls; orthogonal assays; plausible concentrations; target engagement	Baell and Holloway, 2010
Apigenin	Limited compound-specific PAINS evidence; exposure and assay limitations	Assay controls; orthogonal confirmation; plausible exposure; target validation	Capuzzi et al. (2017)

A PAINS, or assay-interference alert is a cautionary indicator, not proof that a compound or all of its reported biological effects are invalid (Capuzzi et al., 2017). Structural studies likewise show that PAINS, classes differ in their binding behavior and may display either genuine or promiscuous interactions (Bolz et al., 2021).

### 4.1 Classification of polyphenols with bioactivity

Polyphenols represent a structurally diverse class of plant secondary metabolites characterized by ≥ 1 aromatic ring bearing −OH substituents (Singla et al., 2019), and are broadly classified into **flavonoids** and **non-flavonoids** based on their core chemical scaffolds (Pandey and Rizvi, 2009). Flavonoids share a conserved C6–C3–C6 backbone and are further subdivided into flavonols (e.g., quercetin), flavones (e.g., luteolin), flavan-3-ols (e.g., catechins), anthocyanins, and flavanones (Maniglia et al., 2021). These are abundant in fruits, vegetables, and beverages such as tea and wine (Bhuyan and Handique, 2022). Structural variations arise from differences in hydroxylation, methylation, glycosylation, and conjugation, which directly influence their redox properties and interactions with target molecules (Tsao, 2010). These metabolites exert potent antioxidant activity and, more crucially, modulate cell signaling pathways implicated in stress responses (Subhan et al., 2024). For example, quercetin upregulates Glo1 and induces Nrf2 nuclear translocation, thereby enhancing MG detoxification and attenuating AGE–RAGE signaling (Meng-Ya et al., 2017). Catechins, particularly EGCG, exhibit similar properties in neuronal and vascular models (Chen et al., 2025; Zhou et al., 2025), indicating that the molecular configuration of flavonoids plays a critical role in determining their activity spectrum.

**FIGURE 2:**
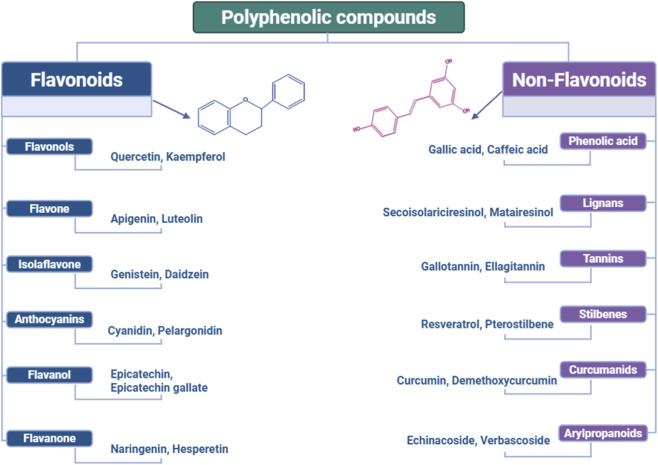
Classification of polyphenolic compounds into flavonoid and non-flavonoid subclasses. This figure summarizes the major structural classes of polyphenols discussed in the review. The classification distinguishes flavonoid and non-flavonoid subclasses and highlights representative compounds relevant to modulation of oxidative stress, glycation, and inflammatory signaling.

Non-flavonoid polyphenols include stilbenes (resveratrol, pterostilbene), phenolic acids (ferulic acid, gallic acid), and lignans. These metabolites are widely distributed in plant-derived foods, including fruits, such as *Vitis vinifera* L., beverages, such as tea (*Camellia sinensis* (L.) Kuntze), and various medicinal plants, where they contribute to plant defense and stress adaptation (Manach et al., 2004). These metabolites also exhibit substantial bioactivity within the Glo1–Nrf2–RAGE framework (Reinisalo et al., 2015). Resveratrol activates SIRT1 and AMPK (Lin et al., 2010), further promoting Nrf2 stability and increasing *Glo1* expression (Cheng et al., 2012; Xue et al., 2025). Similarly, ferulic acid enhances Glo1 activity by modulating redox-sensitive transcription factors, including Nrf2 (Rizvi et al., 2021) and downregulates RAGE expression in models of metabolic syndrome and neuroinflammation (Liu et al., 2018; Neopane et al., 2023). The unique structural motifs of non-flavonoids help target distinct yet complementary pathways during redox regulation and glycation control (Figure 2).

The bioactivities of polyphenols are strongly dependent on their chemical structure, which governs antioxidant capacity, metal-chelating properties, and modulation of cell signaling pathways. Structure–activity relationships are therefore critical for understanding how polyphenols influence redox regulation and inflammatory signaling (Scalbert et al., 2005). Mechanistic findings for some polyphenols, particularly quercetin and curcumin, should be interpreted with caution because PAINS-like behavior can arise from redox cycling, fluorescence interference, aggregation, metal chelation, or nonspecific protein binding (De Matos and Menezes, 2023). Therefore, pathway-specific claims regarding Nrf2, Glo1, or RAGE are strongest when supported by orthogonal assays and *in vivo* or clinical validation.

### 4.2 Modulation of Nrf2 by polyphenols

Nrf2 pathway activation is one of the primary mechanisms by which polyphenols exert their cytoprotection (Islam et al., 2023). Many polyphenols act as indirect electrophiles that modify cysteine residues on Keap1, the protein that ordinarily targets Nrf2 for ubiquitin-mediated degradation (Zhou et al., 2019). This cysteine modification disrupts the Keap1–Cullin3–Nrf2 complex, stabilizing and enhancing the nuclear accumulation of Nrf2 (Dinkova-Kostova et al., 2017). Once in the nucleus, Nrf2 binds to the ARE sequences of genes involved in antioxidant and detoxification responses and initiates their transcription (Dayalan Naidu and Dinkova-Kostova, 2020). Typical examples include curcumin (Liu C. et al., 2023), EGCG (Liu et al., 2022), and quercetin (Lv et al., 2024), which promote this pathway in various cell types under oxidative stress. In addition to modifying Keap1, polyphenols can influence upstream signaling molecules, such as MAPKs and PKC, which phosphorylate Nrf2 and enhance its nuclear translocation (Spencer, 2007; Keskin, 2023). The downstream effects include the upregulation of HO-1, NQO1, and GCLC, collectively reducing ROS levels and restoring redox balance (Li et al., 2014). Importantly, Nrf2 activation also upregulates *Glo1* transcription, linking antioxidant defense to carbonyl-stress detoxification (Xue et al., 2012). The modulation of this pathway by polyphenols is context-specific, with some metabolites preferentially activating Nrf2 in diseased or inflamed cells, thereby minimizing potential off-target effects (Scapagnini et al., 2011). Such a selective sensitivity underscores the therapeutic relevance of polyphenols in managing chronic disorders characterized by dysregulated oxidative signaling.

### 4.3 Induction of Glo1 expression and activity

Beyond antioxidant defense, polyphenols also directly influence the glyoxalase pathway by enhancing *Glo1* expression and enzyme activity (Aragonès et al., 2021). This regulation has been observed in endothelial, neuronal, hepatic, and renal cell models (Delle Monache et al., 2021; Najjar et al., 2022; Tang et al., 2023). Polyphenols such as quercetin, resveratrol, hesperetin, and apigenin upregulate Glo1 at the transcription and translation levels (Yu et al., 2023; Xue et al., 2025; Zheng et al., 2025). These metabolites mainly act by activating Nrf2, AP-1, and HIF-1α (Hussain et al., 2022; Ramezani et al., 2023) as well as interfering with post-translational modifications (Della Vedova et al., 2025). Furthermore, affecting redox-sensitive pathways and cofactors is another key mechanism by which polyphenols influence Glo1 activity (Garcia Cortizo, 2024). By maintaining intracellular GSH levels and enhancing the cell’s antioxidant capacity, polyphenols create a favorable environment for Glo1’s catalytic activity (Wang Y. et al., 2023). In streptozotocin-induced diabetic rat models, hesperetin or hesperidin enhanced Nrf2/ARE/Glo1 signaling and reduced AGE–RAGE-associated oxidative and inflammatory injury (Chen et al., 2019; Zhu et al., 2020).

### 4.4 Inhibition of RAGE expression and ligand binding

**FIGURE 3:**
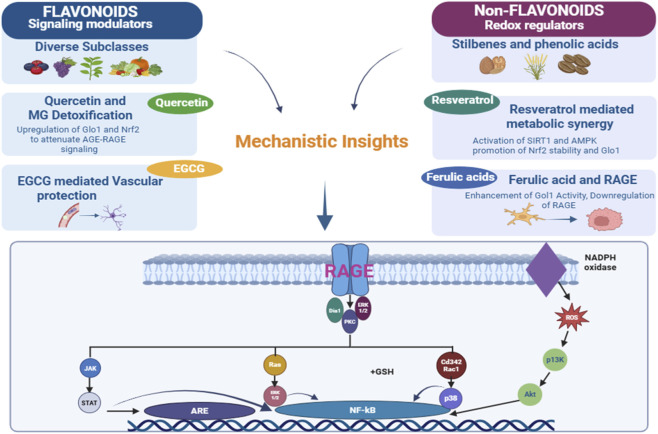
Mechanistic overview of flavonoid and non-flavonoid actions on redox, carbonyl-stress, and RAGE-associated inflammatory signaling. This figure summarizes how representative flavonoids and non-flavonoids may influence the Glo1–Nrf2–RAGE axis. Polyphenols are shown as modulators of methylglyoxal detoxification, Nrf2-linked cytoprotection, and RAGE-associated inflammatory signaling rather than as direct clinical therapeutics.

RAGE inhibition by polyphenols is a key intervention point, as excessive AGE–RAGE interaction drives a cycle of inflammation, oxidative stress, and tissue damage (Li et al., 2023). Polyphenols such as luteolin (Zhang N. et al., 2024), curcumin (Morasso et al., 2024), and chlorogenic acid (He et al., 2024) reduce RAGE mRNA and protein levels in *vitro* and *in vivo* models (Nakagawa, 2022). Because RAGE signaling and NF-κB activation can reinforce each other, suppression of NF-κB- and AP-1-associated inflammatory signaling may reduce downstream RAGE pathway activity; however, this should not be interpreted as direct RAGE transcriptional repression unless RAGE transcription was measured (Lu et al., 2024). Polyphenols may lower AGE–RAGE signaling through several mechanisms, including reduced AGE formation, enhanced Glo1-mediated MGO detoxification, interference with AGE–RAGE interaction, and in some models, lowered RAGE expression. Furthermore, a few polyphenols interfere with direct AGE–RAGE binding, thereby potentially interfering with ligand–receptor interaction. For instance, resveratrol (Jing et al., 2010), quercetin (Yang S. et al., 2020), luteolin (Gao et al., 2025), and a polyphenol-rich diet (Wu et al., 2025) have been used. Computational tools have further predicted that resveratrol and its analogs can occupy the ligand-binding pocket of RAGE, thereby inhibiting AGE-induced signaling cascades (Shi et al., 2023; Muthyalaiah et al., 2024). This mechanism reduces the production of IL-6 (Interleukin-6), TNF-α (Tumor Necrosis Factor-alpha), and ICAM-1 (Intercellular Adhesion Molecule1), particularly in endothelial and immune cells (Shen et al., 2020; Losso, 2022) (Figure 3). Accordingly, the present review distinguishes direct reductions in RAGE expression from indirect attenuation of AGE–RAGE signaling caused by lower MGO burden, reduced AGE formation, or inhibition of ligand–receptor interaction.

### 4.5 Bioavailability and pharmacokinetics

Polyphenols generally exhibit poor oral bioavailability due to limited absorption, extensive first-pass metabolism, and rapid systemic clearance (Losso, 2022; Datta et al., 2026). Once ingested, polyphenols undergo the phase I and II stages of metabolism in the intestinal mucosa and liver, including methylation, sulfation, and glucuronidation, generating a variety of conjugated metabolites with altered bioactivity (Manach and Donovan, 2004). Although sometimes less potent than the parent metabolites, these metabolites can retain or acquire novel functional properties (Batiha et al., 2020) that influence redox and inflammatory pathways. Notably, quercetin glucuronides and methylated catechin derivatives maintain their capacity to induce Nrf2 activation and suppress RAGE expression, albeit with a different potency (O’Leary et al., 2003; Docampo-Palacios et al., 2020).

The gut microbiota’s role in biotransformation is increasingly gaining recognition as a factor influencing polyphenol pharmacokinetics (Wang M. et al., 2023). Microbial metabolism significantly enhances the generation of bioactive derivatives capable of crossing biological barriers, including the blood–brain barrier (Zhang and Davies, 2016; Chen et al., 2022), thereby extending their functional reach to the central nervous system. For example, microbial catabolites of ellagic acid and proanthocyanidins have pronounced effects on Glo1 and Nrf2 in neurodegenerative AD/PD models (Gupta et al., 2021; Zhong et al., 2023). Polyphenols such as EGCG accumulate favorably in neural and hepatic tissues, correlating with their site-specific efficacy (Tang et al., 2021; Zhou et al., 2025). Given these complexities, advancements in nanoformulations, prodrug strategies, and polyphenol-rich dietary interventions are being explored to overcome bioavailability constraints and optimize the systemic modulation of the Glo1–Nrf2–RAGE axis (Yang B. et al., 2020; Hendawy, 2021). The following section applies this axis-based framework to diabetes, where hyperglycemia-driven MG accumulation and AGE–RAGE activation are central pathological features.

## 5 Functional implications in diabetes

Diabetes mellitus, particularly type 2 diabetes mellitus, is characterized by persistent hyperglycemia and metabolic dysregulation of glucose and lipid metabolism (Galicia-Garcia et al., 2020). This disorder is prevalent across the globe, and in 2022, an estimated 828 million adults (18 years and older) had diabetes (Ogurtsova et al., 2022). A disruption of the Glo1–Nrf2–RAGE axis in diabetes is a major driver of co-complications (Wang et al., 2021; Syed et al., 2023), such as nephropathy, retinopathy, neuropathy, and cardiovascular disease (Chen et al., 2019; Tang et al., 2022), making it a critical therapeutic target for disease modulation and prevention of secondary complications.

### 5.1 Hyperglycemia and MG formation

Persistent hyperglycemia increases glycolytic flux, leading to an excessive accumulation of MG and the formation of AGEs (Schalkwijk and Stehouwer, 2020). These glycated macromolecules accumulate within the extracellular matrix and cells, impairing cell function and promoting apoptosis (Kold-Christensen and Johannsen, 2020). In diabetes, enhanced MG production and detoxification capacity do not accompany each other, largely due to Glo1 downregulation (Kold-Christensen and Johannsen, 2020). In both type 1 and type 2 diabetes, Glo1 activity is markedly reduced in the kidney, retina, and vascular tissues (Maessen et al., 2015; Nigro et al., 2017), correlating with elevated MG and AGE contents. Glo1 is suppressed via multiple mechanisms, including oxidative inactivation, reduced gene expression, and altered cofactor availability (glutathione), which are crucial for essential Glo1 function (Xue et al., 2012). Furthermore, in diabetes, oxidative stress induced by MG impairs the Keap1–Nrf2–ARE pathway (Li et al., 2015), limiting the transcription of *Glo1* and other antioxidant-related genes, creating a detrimental feedback loop in which elevated MG levels not only drive AGE formation but also amplify oxidative and inflammatory signaling through sustained RAGE activation (Moreira et al., 2022). The AGE–RAGE interaction exacerbates diabetes-associated complications by promoting endothelial dysfunction (Gao et al., 2008), basement membrane thickening, and inflammatory cell recruitment, which contribute to vascular damage and tissue fibrosis (Rojas and Morales, 2004). These events collectively underscore the centrality of MG accumulation and Glo1 suppression in the molecular pathology of diabetes (Berner et al., 2012; Alhujaily, 2024).

The cardiovascular relevance of the Glo1–Nrf2–RAGE axis is important in diabetes because hyperglycemia-induced methylglyoxal accumulation, AGE formation, oxidative stress, and RAGE activation greatly contribute towards endothelial dysfunction and thromboinflammatory risk (Vulesevic et al., 2016). Polyphenols may attenuate these processes by enhancing Nrf2-dependent antioxidant defense, supporting Glo1-mediated methylglyoxal detoxification, and reducing AGE–RAGE-associated inflammatory signaling (Mohd Nor et al., 2022). Recent evidence further indicates that hydroxytyrosol protects human erythrocytes against hyperglycemia-induced phosphatidylserine exposure by modulating redox balance and calcium homeostasis, suggesting a potential mechanism for limiting prothrombotic alterations in diabetic vascular complications (Notariale et al., 2025).

### 5.2 Nrf2 in diabetic organ protection

Nrf2 plays a pivotal role in defense against oxidative stress and metabolic insults associated with diabetes, primarily by regulating the transcription of genes encoding antioxidation and detoxification-associated enzymes (David et al., 2017; Ali et al., 2026). Under physiological conditions, Nrf2 maintains redox homeostasis and supports cell integrity (Ma, 2013) by activating genes encoding enzymes such as HO-1, NQO1, and glutamate–cysteine ligase (Loboda et al., 2016). However, in diabetes, chronic oxidative stress and hyperglycemia impair Nrf2 nuclear translocation and reduce its transcriptional activity, thereby compromising cell defense (Bitar and Al-Mulla, 2011), contributing to the susceptibility of various organs, particularly the kidneys, eyes and blood vessels to diabetic damage. For instance, in diabetic nephropathy, decreased Nrf2 activity enhances oxidative damage in glomerular endothelial cells and promotes mesangial matrix expansion and proteinuria (Hu et al., 2024). Restoration of Nrf2 activity in diabetic models attenuates these pathological changes. The pharmacological activation or upregulation of Nrf2 via dietary antioxidants restores antioxidant enzyme levels, suppresses inflammatory markers, and protects against tissue fibrosis and apoptosis (Bocci and Valacchi, 2015). In retinal tissues, Nrf2 activation reduces oxidative stress, preserves capillary integrity, and prevents the progression of diabetic retinopathy (Pawlowska et al., 2019; Ouyang et al., 2022). Furthermore, Nrf2 activation also upregulates *Glo1* expression, enhancing MG detoxification and reducing AGE accumulation in diabetic tissues (Frandsen and Narayanasamy, 2017; Nishimoto et al., 2017).

### 5.3 Polyphenol-based modulation in clinical and preclinical studies

Polyphenols have emerged as candidate bioactives that modulate the dysregulated Glo1–Nrf2–RAGE axis in diabetes (Pannucci et al., 2023). To improve interpretability, evidence on polyphenol-mediated modulation of the Glo1–Nrf2–RAGE axis in diabetes has been organized by experimental level.

#### 5.3.1 *In vitro* evidence

Several studies in cultured cells demonstrate that metabolites such as quercetin, hesperidin, and p-coumaric acid reduced RAGE-associated signaling activated by AGE exposure (Takata et al., 2025). In high-glucose or AGE-stimulated cell models, quercetin reduces RAGE expression, NF-κB translocation, and proinflammatory cytokines, suggesting a direct blockade of AGE–RAGE-driven inflammation (Takata et al., 2025). These effects improve cell redox balance and reduce glycation stress in diabetic experimental systems (Groh et al., 2020). Evidence from cell models provides mechanistic insight into how polyphenols modulate the Glo1–Nrf2–RAGE axis under hyperglycemic or carbonyl-stress conditions. In SH-SY5Y cells exposed to chronic high glucose, quercetin activated the Nrf2/ARE pathway and increased Glo1 activity, supporting the role of quercetin in enhancing MG detoxification at the cell level (Liu et al., 2019). In HepG2 cells, resveratrol upregulated Nrf2 expression and attenuated MG-induced insulin resistance, linking Nrf2 activation with protection against carbonyl-stress-related metabolic dysfunction (Cheng et al., 2012).

#### 5.3.2 In vivo pre clinical evidence

In a study of streptozotocin-induced diabetic rats, hesperetin remarkably upregulated Glo1 activity, thereby facilitating detoxification of MG, a major precursor of AGEs. Additionally, Nrf2 activation enhanced antioxidant defenses and reduced RAGE expression, renal fibrosis, and oxidative stress, indicating the potential of hesperetin to ameliorate diabetic nephropathy by modulating the Glo1–Nrf2–RAGE axis (Chen et al., 2019). In the same model, hesperetin activated the Nrf2–ARE–Glo1 pathway, increased *Glo1* expression, and suppressed AGE–RAGE signaling in renal tissues, which was associated with reduced inflammation, oxidative stress, and diabetic nephropathy progression (Chen et al., 2019).

In another study, citrus flavonoids significantly reduce AGE accumulation and RAGE expression in the kidneys of diabetic rats as well as the levels of proinflammatory cytokines, such as IL-1β and TNF-α, and oxidative stress markers. These findings suggest that flavonoids can help regulate the AGE–RAGE pathway and redox balance, thereby associated with a protective influence in preclinical models against diabetic complications, including nephropathy (Xiao et al., 2024). During an investigation of diabetic kidney disease, hesperetin treatment elevated *Glo1* expression, thereby reducing AGE-induced damage. The activation of Nrf2 further facilitated cell defense mechanisms, reducing inflammation and fibrosis in kidney tissues. These results highlight the role of hesperetin in regulating the Glo1–Nrf2–RAGE axis, providing a potential therapeutic approach for diabetic kidney disease (Efiong et al., 2025).

In a small human intervention study, acute consumption of bilberry (*Vaccinium myrtillus* L.) extract significantly modulated the transcription of Nrf2-regulated antioxidant-associated genes in peripheral blood cells. After a polyphenol-reduced diet, five healthy male subjects consumed a bolus (700 mL) of respective test beverages with blood sampling for up to 8 h after intake. All beverages affected Nrf2, HO-1, and NQO-1 transcription with different potencies and durations (Groh et al., 2020). However, a limited sample size, absence of long-term follow-up, and lack of clinical endpoints restricted the interpretation of these findings to preliminary, hypothesis-generating evidence rather than demonstrating clinical efficacy.

A major translational limitation is the difference between polyphenol concentrations used in experimental systems and those achievable in humans. Many *in vitro* studies use micromolar concentrations of parent polyphenols, whereas oral intake in humans often produces nanomolar to low-micromolar plasma concentrations because of limited absorption, rapid phase II metabolism, and extensive conjugation. Moreover, circulating metabolites may differ structurally and functionally from the parent metabolites used in cell experiments. Therefore, findings from cell and animal models should be interpreted as mechanistic or preclinical evidence rather than direct evidence of clinical efficacy.

#### 5.3.3 Clinical evidence

In a randomized clinical trial, tRES-HESP supplementation in overweight and obese individuals with insulin resistance was associated with marked increases in Glo1 activity and a reduction in MG levels, improved insulin sensitivity and glycemic control, and reduced systemic inflammation, as measured by TNF-α and CRP levels (Rabbani et al., 2021). These findings provide preliminary human evidence that polyphenol-based interventions may influence the Glo1–Nrf2–RAGE axis. However the limited number of trials, heterogeneity in formulation, and reliance on surrogate endpoints preclude firm conclusions regarding clinical efficacy. While these findings support translational relevance, interpretation should consider moderate sample size, intervention duration, and population specificity, which may limit generalizability. In another clinical investigation, “GlucoRegulate,” a supplement containing trans-resveratrol and hesperetin, increased Nrf2 activation and upregulated Glo1 in human endothelial and liver cells. This increase in the expression of antioxidant-related genes was accompanied by a decline in the levels of RAGE protein, indicating a suppression of AGE-induced inflammatory responses. This study further suggested that polyphenol supplementation could be an effective approach to managing diabetes-related complications by modulating the Glo1–Nrf2–RAGE pathway (Xue et al., 2025).

In another clinical trial involving patients with type 2 diabetes, supplementation with an EGCG-rich green tea extract (*Camellia sinensis* L.) at 300–900 mg/day significantly increased soluble RAGE (sRAGE) levels in circulation. This RAGE decoy is thought to inhibit AGE–RAGE signaling by competing with membrane RAGE ligands, thereby potentially reducing proinflammatory responses driven by the AGE–RAGE axis in diabetes. Such an effect was mediated by ADAM10-dependent ectodomain shedding, a mechanism by which EGCG can suppress active RAGE signaling, linking polyphenols to a direct modulation of RAGE in humans (Huang et al., 2013). Researchers have also highlighted mechanistic insights into Nrf2-based regulation of Glo1 in diabetes models (González et al., 2020): the Nrf2 pathway plays a crucial role in regulating *Glo1* expression across various diabetes models. Polyphenols, such as hesperetin and resveratrol, can activate Nrf2, thereby enhancing Glo1-mediated detoxification of metabolites like MG. This molecular mechanism explains how polyphenols reduce AGE accumulation, oxidative stress, and inflammation in diabetic tissues (González et al., 2020) (Table 2). Overall, current evidence in humans remains limited and heterogeneous and does not yet establish a consistent clinical efficacy of polyphenols in modulating the Glo1–Nrf2–RAGE axis. More broadly, a translation of polyphenol efficacy from preclinical models to humans remains limited by bioavailability, metabolic variability, and inconsistent clinical trial design (Manach et al., 2004; Di Lorenzo et al., 2021).

**TABLE 2:** A summarized overview of *in vitro, in vivo,* and preclinical studies examining the roles of polyphenols in diabetes.

Study	Polyphenol(s)	System/Model	Glo1/Nrf2/RAGE/AGE findings	Major outcomes	Ref
*In Vitro*	Quercetin	Endothelial cells exposed to high glucose or AGE stress	↑Glo1; ↓AGE; ↑Nrf2/ARE; ↓RAGE/NF-κB	Reduced ROS, inflammation, and apoptosis	Zaman and Amin (2025)
*In Vitro*	EGCG	Endothelial cells cultured in high-glucose concentrations	Inhibits AGE formation and decreases RAGE expression	Preserved endothelial integrity	Martati et al. (2026)
*In Vivo* (Rodent diabetes)	Hesperetin/hesperidin	STZ-induced diabetic rats	↑Nrf2, Glo; ↓AGE/RAGE signaling; ↓ IL-1β, TNF-α	Reduced renal injury and oxidative stress	Xiao et al. (2024)
*In Vivo* (Rodent)	Resveratrol	STZ or HFD diabetes	↑Nrf2; ↓RAGE expression	Improved insulin sensitivity and vascular function	Khazaei et al. (2016), Zhang et al. (2021)
*In Vivo* (Rodent)	Protocatechualdehyde	Diabetic cataract model	↑Glo1; ↓ AGE–RAGE	Reduced lens oxidative damage	Cheng et al. (2025)
Preclinical mixed	Plant phenolics	Diabetic animal models	Modulates AGE–RAGE; Nrf2 pathway	Reduced diabetic tissue complications	Gonzalez et al. (2020)
Clinical (Human metabolic)	Resveratrol + hesperetin (tRES + HESP)	Overweight/obese humans (clinical study)	↑Glo1; ↓plasma MGO; ↓ICAM1	Improved glycemic and inflammatory markers	Xue et al. (2025)
Clinical (T2D interventions)	Polyphenol-rich diets	T2D subjects	Associations with reduced oxidative stress markers	Improved metabolic and inflammatory markers	Coelho et al. (2023), Xue et al. (2025)

Another critical limitation arises from the disparity between concentrations used in experimental systems and those achievable *in vivo*. Many *in vitro* studies employed polyphenols at a micromolar range; however, following oral administration in humans, plasma concentrations are typically in the nanomolar to low-micromolar range due to limited absorption, rapid metabolism, and extensive conjugation. Such a discrepancy complicates the direct extrapolation of mechanistic findings to clinical efficacy and underscores the need to interpret cell data within a pharmacokinetic context.

## 6 Functional implications in neurodegeneration

Neurodegenerative disorders, such as Alzheimer’s disease (AD) and Parkinson’s disease (PD), are progressive, multifactorial conditions (Walker and Attems, 2024). These are generally characterized by oxidative stress, mitochondrial dysfunction, and chronic neuroinflammation (Mankovska et al., 2025). A common molecular hallmark of these pathologies is the overaccumulation of reactive dicarbonyls, particularly MG, leading to the synthesis of AGEs (Li, 2023). These metabolites exert neurotoxicity by crosslinking proteins, damaging mitochondrial membranes, impairing enzyme function, and activating proinflammatory signaling through RAGEs (Schalkwijk and Stehouwer, 2020). In parallel, the detoxification capacity of Glo1 is significantly compromised in aging and neurodegenerative brains (Jiang et al., 2018), further promoting MG-induced glycation and oxidative damage. Concomitantly, Nrf2 is downregulated in neurodegenerative diseases, thereby weakening cell defense mechanisms against oxidative and carbonyl stress (Ramsey et al., 2007).

### 6.1 Carbonyl stress in AD and PD

Accumulation of MG and AGEs in neuronal tissues has been consistently documented in AD and PD, where they were implicated in synaptic dysfunction, tau hyperphosphorylation, and α-synuclein aggregation (Guerrero et al., 2013; Haddad et al., 2019). Post-mortem analyses of AD brains reveal prominent elevations in MG-modified proteins and AGEs within the hippocampus and cerebral cortex, regions critical for memory and cognitive function (Gao, 2008; Prasad, 2019). Similarly, in the PD substantia nigra, MG levels were elevated, correlating with dopaminergic neuronal loss and increased oxidative stress-related markers (Werner et al., 2008; Xie et al., 2014). The accumulation of these glycating agents disrupts proteostasis and enhances mitochondrial fragmentation, thereby initiating neuronal apoptosis and neurodegeneration (Hipkiss, 2017; Zhang et al., 2018). The pathological consequences of AGE accretion are largely mediated through RAGE, which is overexpressed in microglia, astrocytes, and endothelial cells of the neurodegenerative brain (Cai et al., 2016). Engagement of AGEs with RAGE activates downstream signaling cascades, including NF-κB, MAPK, and JAK/STAT pathways (C Tobon-Velasco et al., 2014), thereby promoting the release of proinflammatory cytokines such as IL-1β, IL-6, and TNF-α (Ng et al., 2018), a neuroinflammatory milieu which contributes to synaptic degradation and impairs neurogenesis (Allan and Rothwell, 2001). Moreover, RAGE activation in endothelial cells of the blood–brain barrier increases permeability and facilitates the infiltration of peripheral immune cells into the central nervous system (CNS), thereby exacerbating neurodegenerative processes (Huang et al., 2021). These interconnected mechanisms illustrate how MG accumulation and AGE–RAGE signaling constitute a critical axis that promotes chronic neuroinflammation and oxidative damage, ultimately leading to neuronal death in AD and PD.

### 6.2 Nrf2–Glo1 regulation in brain health

The Nrf2–Glo1 axis preserves neuronal function and redox stability under conditions of oxidative and carbonyl stress (Hara et al., 2021). Nrf2 activation enhances the transcription of *Glo1*, and other phase II detoxifying and antioxidative enzymes, thereby improving MG clearance and reducing the AGE burden in the CNS. In healthy brains, this axis supports synaptic plasticity, maintains mitochondrial function, and limits microglial activation (Navarro and Esteras, 2024). However, in neurodegenerative diseases, Nrf2 expression and nuclear translocation are markedly reduced, particularly within the cortical neurons and hippocampi (Zgorzynska et al., 2021). Such an impairment compromises the transcriptional activation of *Glo1* and other cytoprotective genes, rendering neurons vulnerable to MG-induced toxicity and AGE accumulation (Yang et al., 2022; Alhujaily, 2024). The resultant deficiency in glyoxalase activity has been observed in AD and PD models, contributing to elevated MG levels and sustained RAGE activation.

### 6.3 Clinical and preclinical evidence

Polyphenols have been investigated as neuroprotective agents in experimental models of neurodegeneration, but the extent of evidence for Glo1–Nrf2–RAGE modulation differs among studies. Many polyphenols, including resveratrol, curcumin, EGCG, and apigenin, can cross the blood–brain barrier and exert effects localized to the neural tissues (Grabska-Kobyłecka et al., 2023). In several neurodegeneration models, these metabolites activate Nrf2 and increase antioxidant enzymes such as HO-1 and NQO1. However, direct evidence for Glo1 induction, methylglyoxal clearance, or RAGE suppression should be interpreted only where these endpoints were measured (Bayazid and Lim, 2022; Moratilla-Rivera et al., 2023; Chen et al., 2025). For instance, resveratrol has been reported to improve oxidative-stress and mitochondrial endpoints in AD-related models, mainly through Nrf2-linked antioxidant signaling. These findings support partial-axis modulation unless Glo1, methylglyoxal, AGE, or RAGE endpoints were directly assessed (Tosatti et al., 2022). Similarly, EGCG improves mitochondrial efficiency and reduces ROS production in the dopaminergic neurons of PD models, effects that are partly mediated through Glo1 upregulation and Nrf2 activation (Tosatti et al., 2022). Moreover, polyphenols, including flavonoids and terpenoids, support neurogenesis and synaptic remodeling by enhancing the contents of neurotrophic factors, such as brain-derived neurotrophic factor, thereby contributing to a functional recovery in neurodegenerative AD/PD models (Amidfar et al., 2024).

Numerous preclinical experiments have shown that resveratrol confers neuroprotection in rodent models of AD by activating the Nrf2-dependent antioxidant pathway, increasing the expression of downstream antioxidant genes and mitochondrial function, which combats oxidative damage central to AD pathology (Islam et al., 2022; Bartra et al., 2024). Because not all neurodegeneration studies measured Glo1, methylglyoxal, AGE accumulation, or RAGE, Nrf2 activation alone was interpreted as partial-axis evidence. Such findings indicate antioxidant pathway modulation but do not establish complete regulation of the Glo1–Nrf2–RAGE axis (Naomi et al., 2023). A study reported the therapeutic potential of baicalin against diabetes-associated cognitive impairment (DCI) in GK rats and db/db mice, by improving cognitive function, reducing Aβ and phosphorylated Tau levels, and alleviating neuronal damage. Bai’s neuroprotective influence was linked to antioxidant and anti-inflammatory actions, which Nrf2-blocker-based experiments confirm to be mediated through the KEAP1-Nrf2 signaling pathway. These findings suggest that Bai could serve as a promising therapeutic strategy for DCI by modulating the KEAP1-Nrf2 pathways (Zheng et al., 2024).

Several preclinical studies (*in vivo and in vivo*) have shown quercetin and other flavonoids to possess neuroprotective actions in models of PD and AD, including reduced neuroinflammation, suppression of oxidative stress, and improved neuronal survival. These effects are linked to the enhancement of endogenous antioxidant systems via Nrf2 activation and diminished aggregation of neurotoxic proteins. By attenuating oxidative damage and related inflammatory signaling, these polyphenols indirectly reduce AGE–RAGE-mediated injury in neuronal tissues, suggesting a beneficial modulation of this axis under neurodegenerative settings (Zhang L. et al., 2024). Likewise, mangiferin, a natural polyphenolic xanthone, upregulates Glo1 in central neurons cultured under high-glucose conditions by activating the Nrf2–ARE signaling pathway. In hippocampal and cortical neurons, high-glucose downregulates Glo1 and Nrf2 nuclear translocation, increases AGE formation, and oxidative stress. Co-treatment with mangiferin restored Glo1 expression and activity and enhanced Nrf2/ARE target genes, thereby elevating glutathione levels and reducing AGE accumulation, indicating a mechanistic link between a polyphenolic metabolites and Glo1/Nrf2 modulation in a neuronal context (Liu et al., 2017). Simultaneously, Xue et al. (2025) examined the effects of a trans-resveratrol and hesperetin formulation (tRES + HESP) in primary human endothelial cells, primary human fibroblasts, and HepG2 hepatoma cells. It increased *Glo1* expression and multiple Nrf2/ARE-linked genes at the mRNA and protein levels, reflecting an activation of the Nrf2 transcriptional responses; RAGE protein levels were reduced, consistent with decreased AGE/RAGE signaling (Xue et al., 2025). In a study involving streptozotocin-induced diabetic rats, chronic hesperidin administration improved diabetes-associated behavioral deficits and oxidative stress in the brain. Hesperidin treatment was associated with higher Nrf2 protein levels, upregulation of γ-glutamylcysteine synthetase, restoration of Glo1 expression, and attenuation of AGE/RAGE signaling and oxidative stress markers in the brain, as reported in the cited study. Crucially, using an Nrf2 inhibitor reversed these benefits, showing that such neuroprotection was mediated through Nrf2/ARE → Glo1 regulation and reduced AGE/RAGE signaling (Zhu et al., 2020) (Table 3).

**TABLE 3:** An overview of *in vitro, in vivo,* and preclinical studies using polyphenols to treat neurodegenerative diseases.

Model/Study type	Polyphenol(s)	System/Model	Glo1/Nrf2/RAGE/AGE findings	Major outcomes	References
*In Vitro* (Neurodegenerative) ation)	Quercetin	SH-SY5Y cells + chronic high glucose	↑Nrf2/ARE; ↑Glo1; ↓AGE accumulation	Reduced ROS, inflammation, and apoptosis	Liu et al. (2019)
*In Vitro* (Neurodegenerative) ation)	Quercetin	SH-SY5Y + methylglyoxal	ROS levels; apoptosis	Reduced MG-induced ROS and apoptosis	Akol and Taşkıran (2024)
*In Vitro* (Oxidative Stress)	General flavonoids	Cell models	Nrf2 activation; Glo1 induction	Induced antioxidant and glyoxalase-related genes	Frandsen and Narayanasamy (2018)
*In Vivo* (Neurodegenerative) ation)	Mangiferin	Rat TBI model	↓ Oxidative stress and inflammatory mediators	Reduced brain oxidative damage and inflammation	Mustafa et al. (2025)
*In Vivo* (Systemic Glo1 Induction)	Resveratrol + hesperetin coformulation	Human PBMC Glo1 activity (obese subjects)	↑Glo1 activity	Increased PBMC Glo1 activity	Xue et al. (2016)
*In Vivo* (Diabetes)	Hesperetin/hesperidin	STZ-induced diabetic rats	↑Nrf2 and Glo1 expression; ↓AGE, RAGE	Reduced renal injury and AGE–RAGE signaling	Xiao et al. (2024)
Cell model	Resveratrol	BV2 microglia + monomeric C-reactive protein	↑ Sirt1/Nfe2L2-linked antioxidant signaling; ↓ NF-κB inflammatory signaling	Reduced oxidative stress	Bartra et al. (2024)
*In Vivo* (Mice)	Protocatechualdehyde	Parkinson’s Model	↑Glo1; ↓ AGE–RAGE	Reduced oxidative stress	​
*In Vivo* (Mice)	Flavonoids	Glo1 model, Parkinson’s disease	↑Glo1; ↓plasma MGO; ↓ ICAM1	Protected against neurodegenerative injury	Frandsen and Narayanasamy (2017)
*In vivo* (rat)	Quercetin	Diabetic rat/brain	↓ oxidative stress markers	Improved metabolic and inflammatory markers	Zhu et al. (2018)

AGE, advanced glycation end product; ARE, antioxidant response element; EGCG, epigallocatechin gallate; Glo1, glyoxalase 1; HFD, high-fat diet; ICAM-1, intercellular adhesion molecule 1; NF-κB, nuclear factor-κB; Nrf2, nuclear factor erythroid 2–related factor 2; RAGE, receptor for advanced glycation end products; ROS, reactive oxygen species; STZ, streptozotocin; T2D, type 2 diabetes. ↑ indicates increased and; ↓ indicates decreased expression, activity, level, or pathway activation relative to the corresponding control group as reported in the cited study.

## 7 Challenges, limitations, and future prospects

Despite compelling evidence supporting the modulatory effects of polyphenols on the Glo1–Nrf2–RAGE axis, their successful translation into clinical applications remains constrained by several scientific, pharmacokinetic, and regulatory limitations. The complexity of polyphenol metabolism, variability in bioavailability, and individual-specific responses significantly impact their efficacy *in vivo* (Cladis et al., 2022).

### 7.1 Translational challenges, safety, and future directions

One of the most persistent limitations in polyphenol research is the discrepancy between robust preclinical outcomes and modest or inconsistent clinical efficacy (Aatif, 2023). Most *in vitro and in vivo* studies are conducted using relatively high concentrations of isolated polyphenols or their aglycones, often exceeding levels physiologically attainable by human tissues (Di Lorenzo et al., 2021; Saad et al., 2025). The pharmacokinetic profiles of polyphenols are highly variable due to factors such as gastrointestinal metabolism, enzyme degradation, and hepatic conjugation (Rahman et al., 2021; Lippolis et al., 2023). Collectively, these reduce the concentration of the parent metabolites of such bioactives that reach the systemic circulation. Additionally, the *in vivo* polyphenol metabolites differ significantly from their original structures, often demonstrating altered bioactivity or target affinity (Stromsnes et al., 2021). These factors confound a direct extrapolation of experimental data to clinical scenarios.

Furthermore, human trials investigating polyphenol efficacy frequently lack standardization in terms of formulation, dosage, duration, and endpoint selection (Stromsnes et al., 2021). Heterogeneity among patient populations, including genetic polymorphisms regarding detoxification enzymes, gut microbiota composition, dietary patterns, and underlying pathophysiology, further obscures the interpretation of trial outcomes (Stromsnes et al., 2021; Muijsenberg et al., 2026). In diseases such as diabetes and neurodegeneration, where progression is slow and multifactorial, identifying short-term biomarkers that reliably reflect long-term clinical benefit remains a challenge (Cummings et al., 2025). Without rigorous pharmacodynamic and pharmacokinetic profiling, it is hard to establish dose–response relationships or determine the minimal effective polyphenolic-rich concentrations required to modulate the Glo1–Nrf2–RAGE axis in humans. Addressing these limitations will require harmonized trial designs, validated biomarkers of redox and carbonyl stress, and placing greater emphasis on formulation optimization.

### 7.2 Nano delivery and formulation of polyphenols

The intrinsic limitations of polyphenol-based drug delivery are being addressed using advanced nanotechnology. Various nanoformulations, including micelles, liposomes, solid lipid nanoparticles, and polymeric nanocarriers, have markedly improved the pharmacokinetic profiles of drug moieties (Rudrapal et al., 2022; Ranjbar et al., 2023). Designing polyphenol-based nanoformulations for precise targeted delivery and protection against gut microbiota is highly beneficial. Various investigations have produced promising results; for instance, curcumin-loaded liposomes and EGCG-encapsulated nanospheres (Jin et al., 2022; Asiri et al., 2025), and tea polyphenols encapsulated as nanoformulations (Niu et al., 2022; Ahmadi et al., 2025) demonstrated enhanced bioavailability and sustained release. These delivery systems also enable co-encapsulation of multiple polyphenols and their combination with synthetic antioxidants or chemotherapeutics. The limited bioavailability of polyphenols represents a major constraint for clinical translation. Following oral intake, most polyphenols undergo extensive phase II metabolism, resulting in circulating concentrations that are substantially lower than those used in many *in vitro* studies (Manach et al., 2004). Nanoformulation strategies, including liposomes, polymeric nanoparticles, and self-emulsifying systems, have been developed to enhance stability, absorption, and systemic exposure. However, current evidence does not yet demonstrate that such approaches consistently achieve pharmacologically relevant concentrations in target human tissues (D'Archivio et al., 2007).

A recent nanotechnological advancement is the targeting of ligands that can recognize overexpressed receptors in disease tissues (Khan et al., 2022), such as RAGE in the inflamed vasculature (Woźniak et al., 2021) or tumor microenvironments (Prajapati et al., 2024). This strategy can minimize off-target effects and systemic toxicity as well as increase therapeutic precision. Moreover, site-specific drug delivery can also be achieved by developing nanocarriers responsive to stimuli: pH, redox gradients, or enzymatic activity. Such polyphenol-based formulations have been successfully used in several pathological niches, such as cancer, diabetes, and other disorders (Tie and Tan, 2022; Liu Q. et al., 2023) characterized by high oxidative or glycation stress. Nevertheless, regulatory complexities, scalability challenges, and the safety of nanoformulations remain challenges despite enormous advances (Kardani, 2024; Lu et al., 2025). Therefore, ongoing efforts must focus on standardizing nanoformulation techniques, validating their biocompatibility, and integrating them into well-designed clinical trials.

### 7.3 Influence of personalized nutrition and epigenetics

In modern medicine, polyphenol-based personalized nutrition presents a new Frontier in the context of interindividual variability in metabolic responses, gut microbial composition, and genetic determinants of redox and detoxification pathways (Zhang et al., 2022). Here, polymorphisms in genes encoding key enzymes like Glo1 and Nrf2 (NFE2L2) greatly affect the Glo1–Nrf2–RAGE axis (Kalapos et al., 2022; Cely et al., 2026). To explore this effect, various “nutrigenomic” investigations are in progress, which have revealed the interactions of genetic variants with dietary metabolites to alter cell redox status and glycation load (Kiani et al., 2022). Similarly, the role of various other factors, including epigenetic modifications, histone acetylation, DNA methylation, and miRNA expression profiles, is crucial as a regulatory layer (Zhang et al., 2022). These are essential in shaping the transcriptional activity of Glo1, Nrf2, and RAGE (Kowluru, 2023). Polyphenols may support stress adaptation by modulating DNA methylation, histone modifications, and microRNA expression within the Keap1–Nrf2 regulatory network, thereby increasing Nrf2-dependent antioxidant and detoxification responses, including Glo1-linked methylglyoxal clearance (Frandsen and Narayanasamy, 2018; Bhattacharjee and Dashwood, 2020).

The metabolic fate of nutraceuticals, including polyphenolics, is largely determined by the gut microbiota (Bié et al., 2023). These microbial communities generate catabolites that influence the systemic bioactivity of polyphenols, thereby affecting permeability across biological barriers (Lu et al., 2021). Thus, the pharmacodynamics of polyphenols are affected by individual-specific microbial profiles and offer opportunities for microbiome-targeted dietary interventions (Plamada and Vodnar, 2021). By integrating genomic, epigenomic, and microbiomic data, precision nutraceutical strategies can be developed to optimize the efficacy of polyphenols in disease prevention and management.

### 7.4 Safety, dosing, and translational considerations

Although polyphenols can modulate the Glo1–Nrf2–RAGE axis, their therapeutic translation requires a careful evaluation of dose, route of administration, treatment duration, model system, sample size, and endpoint selection. Preclinical studies frequently use concentrations that exceed physiologically achievable levels, limiting direct extrapolation to humans (Manach et al., 2004;Di Lorenzo et al., 2021). Clinical evidence remains heterogeneous, with variability in formulation, study design, and population characteristics influencing outcomes (D’Archivio et al., 2010).

Safety considerations are particularly relevant for high-dose supplementation and advanced delivery systems. While polyphenols are generally well tolerated at dietary levels, concentrated formulations may induce adverse effects, including gastrointestinal disturbances, adverse modulation of drug-metabolizing enzymes, and hepatotoxicity, as was reported for high-dose EGCG (Additives et al., 2018; Hu et al., 2018). Nanoformulation strategies may enhance bioavailability and tissue targeting; however, they introduce formulation-dependent risks, such as altered biodistribution, tissue accumulation, and insufficiently characterized long-term toxicity (Yang B. et al., 2020).

### 7.5 PAINS and implications for polyphenol pharmacology

An additional limitation concerns the potential pan-assay interference compound (PAINS)-like behavior of several polyphenols. Metabolites such as quercetin and curcumin-like scaffolds may produce nonspecific or false-positive signals through redox cycling, aggregation, metal chelation, fluorescence interference, membrane perturbation, or promiscuous protein binding (Baell and Holloway, 2010). This issue is particularly relevant when mechanistic claims are derived mainly from single *in vitro* assays or from concentrations that exceed physiologically achievable levels. Structural analyses further indicate that PAINS-related promiscuity may arise from diverse binding modes rather than true target selectivity (Bolz et al., 2021).

Accordingly, this review does not treat *in vitro* modulation of Nrf2, Glo1, RAGE, or AGE-related endpoints as evidence of pharmacological or clinical relevance by itself. Such findings are interpreted as mechanistic or hypothesis-generating unless supported by orthogonal assays, counter-screening for assay interference, concentration–response validation within physiologically plausible ranges, metabolite-based testing, target-engagement studies, and *in vivo* or clinical confirmation (Nelson et al., 2017). This distinction is important because PAINS alerts do not necessarily invalidate all biological observations, but they substantially lower confidence in target-specific claims when validation is limited (Dirimanov and Högger, 2020).

## 8 Conclusion

The role of the Glo1–Nrf2–RAGE axis in maintaining cellular redox and carbonyl balance is critical in several disease conditions like diabetes and neurodegeneration. The review underlines the mechanistic insights into “how impairments in glyoxalase activity, antioxidant signaling, and persistent AGE–RAGE interactions” drive pathophysiology. Polyphenols are key bioactive molecules that can critically influence the Glo1–Nrf2–RAGE axis. Their diverse mechanisms of action, including antioxidant effects and modulation of cell stress responses, position them as potential candidates for targeted nutraceutical interventions. Polyphenols have demonstrated bioactivity across cell and animal models, while clinical evidence remains heterogeneous and context-dependent, due to variability in study design, formulation, and measured endpoints. They also affect mitochondrial function and metabolic adaptation, underscoring their relevance to disease modification. Advances in formulation technologies, such as nanocarriers and targeted delivery systems, address challenges in the bioavailability and tissue-specific action. Personalized approaches that account for genetic factors and interactions among microbiota can enhance the therapeutic efficacy of polyphenols. They offer a promising strategy to combat oxidative and glycation stress across a range of age-related and chronic conditions. Although the evidence supports mechanistic plausibility, it does not yet establish consistent clinical efficacy, highlighting the need for well-designed, adequately powered human studies. Future polyphenol-based applications must focus on personalized, evidence-based approaches for long-term disease management and prevention. One of the key limitations of this review is the heterogeneity of the evidence base, spanning *in vitro*, animal, and limited clinical studies with variability in design, dose, and endpoints. Clinical data remain limited and often rely on small sample sizes or surrogate outcomes. In addition, variability in polyphenol bioavailability and formulation complicates cross-study comparison. Consequently, current evidence supports mechanistic plausibility but does not establish consistent clinical efficacy or standardized dosing.

## Supplementary material

Supplementary Table S1 Taxonomic validation of plant species and plant-derived preparations cited in the review.
