# Supplementary material for: Polyphenol-based modulation of the Glo1–Nrf2–RAGE axis in diabetes and neurodegeneration: mechanistic evidence, translational constraints, and critical appraisal
Source: Front Pharmacol. 2026 Jul 20;17:1877182. doi: 10.3389/fphar.2026.1877182 (PMC13430338; doi:10.3389/fphar.2026.1877182)
Supplement: Supplementary file 1 [file Table1.docx]

**Supplementary data**

Table S1. Taxonomic validation of plant species and plant-derived preparations cited in the review

| Common/source name | **Accepted species name with authority** | **Family** | **Plant part/preparation** | **Pharmacopoeial drug name** | **Validation source** |
| --- | --- | --- | --- | --- | --- |
| Green tea / EGCG | Camellia sinensis (L.) Kuntze | Theaceae | Leaf; green tea extract; EGCG-rich preparation | To be checked in relevant pharmacopoeia/MPNS | MPNS/POWO |
| Grape / resveratrol | Vitis vinifera L. | Vitaceae | Fruit/skin-derived stilbene source | To be checked in relevant pharmacopoeia/MPNS | MPNS/POWO |
| Bilberry extract | Vaccinium myrtillus L. | Ericaceae | Fruit extract | To be checked in relevant pharmacopoeia/MPNS | MPNS/POWO |
| Turmeric / curcumin | Curcuma longa L. | Zingiberaceae | Rhizome; curcuminoid preparation | To be checked in relevant pharmacopoeia/MPNS | MPNS/POWO |
| Citrus flavanones / hesperidin-hesperetin | Species should be specified from the cited study | Rutaceae | Fruit peel/flavanone preparation | To be checked in relevant pharmacopoeia/MPNS | MPNS/POWO |
